# Antibiotic Resistance and Virulence Gene Characteristics of Methicillin-Resistant *Staphylococcus aureus* (MRSA) Isolated from Healthy Edible Marine Fish

**DOI:** 10.1155/2020/9803903

**Published:** 2020-06-04

**Authors:** Justine Fri, Henry A. Njom, Collins N. Ateba, Roland N. Ndip

**Affiliations:** ^1^Department of Microbiology, Faculty of Natural and Agricultural Sciences, North-West University, Private Bag X2046, Mmabatho 2735, South Africa; ^2^Agricultural Research Council, Private Bag X1251, Potchefstroom 2531, South Africa; ^3^Department of Microbiology and Parasitology, Faculty of Science, University of Buea, P.O. Box 63, Buea, Cameroon

## Abstract

Thirty-three (33) isolates of methicillin-resistant *Staphylococcus aureus* (MRSA) from healthy edible marine fish harvested from two aquaculture settings and the Kariega estuary, South Africa, were characterised in this study. The phenotypic antimicrobial susceptibility profiles to 13 antibiotics were determined, and their antibiotic resistance determinants were assessed. A multiplex PCR was used to determine the epidemiological groups based on the type of SCC*mec* carriage followed by the detection of staphylococcal enterotoxin-encoding genes *sea*-*sed* and the Panton Valentine leucocidin gene (*pvl*). A high antibiotic resistance percentage (67–81%) was observed for Erythromycin, Ampicillin, Rifampicin, and Clindamycin, while maximum susceptibility to Chloramphenicol (100%), Imipenem (100%), and Ciprofloxacin (94%) was recorded. Nineteen (58%) of the MRSA strains had Vancomycin MICs of ≤2 *μ*g/mL, 4 (12%) with MICs ranging from 4–8 *μ*g/mL, and 10 (30%) with values ≥16 *μ*g/mL. Overall, 27 (82%) isolates were multidrug-resistant (MDR) with Erythromycin-Ampicillin-Rifampicin-Clindamycin (E-AMP-RIP-CD) found to be the dominant antibiotic-resistance phenotype observed in 4 isolates. Resistance genes such as *tetM*, *tetA*, *ermB*, *blaZ,* and *femA* were detected in two or more resistant strains. A total of 19 (58%) MRSA strains possessed SCC*mec* types I, II, or III elements, characteristic of healthcare-associated MRSA (HA-MRSA), while 10 (30%) isolates displayed SCC*mec* type IVc, characteristic of community-associated MRSA (CA-MRSA). Six (18%) of the multidrug-resistant strains of MRSA were enterotoxigenic, harbouring the *see, sea,* or *sec* genes. A prevalence of 18% (6/33) was also recorded for the *luk-PVL* gene. The findings of this study showed that marine fish contained MDR-MRSA strains that harbour SCC*mec* types, characteristic of either HA-MRSA or CA-MRSA, but with a low prevalence of enterotoxin and *pvl* genes. Thus, there is a need for continuous monitoring and implementation of better control strategies within the food chain to minimise contamination of fish with MDR-MRSA and the ultimate spread of the bug.

## 1. Introduction

Methicillin-resistant *Staphylococcus aureus* (MRSA) is a major opportunistic pathogen known to cause severe multidrug-resistant infections in animals and humans. It is also a significant foodborne pathogen due to its staphylococcal enterotoxin (SE) producing abilities. These strains result from *S. aureus* that acquire the *mecA* gene carried on an integrated staphylococcal cassette chromosome *mec* (SCC*mec*). The gene encodes a 78-kDa Penicillin-binding protein (PBP2a) with a lower affinity to Methicillin and other beta-lactam antibiotics [1].

Clinicians often face a challenge in treating MRSA infections due to their marked resistance to various classes of antibiotics. The success of this pathogen is related to the remarkable ability of *S. aureus* to quickly adapt and acquire resistance to multiple antibiotics introduced in clinical practice over the years, coupled with its extensive battery of virulence factors [2]. Infections caused by *S. aureus* vary in severity from minor skin infections, such as boils, carbuncles, pimples, abscesses, cellulitis, folliculitis, impetigo, and scalded skin syndrome, to life-threatening conditions, such as bacteraemia, endocarditis, meningitis, toxic shock syndrome, pneumonia, and osteomyelitis. More than 80% of mortality rate was recorded for life-threatening infections caused by the pathogen until the development of Penicillin in the 1940s, which significantly improved therapy [3, 4]. Unfortunately, this medical success was short-lived as nosocomial infections were associated with increased frequency of resistance to Penicillin conferred by the production of a plasmid-located beta-lactamase gene, *bla*Z. The introduction of the first semisynthetic Penicillin, Methicillin, in 1959, later proved ineffective as there also emerged hospital-associated MRSA (HA-MRSA), mostly limited to health care settings [4]. MRSA was later disseminated to the community with the first virulent community-associated MRSA (CA-MRSA) reported in the late 1990s, which possessed the virulent Panton Valentine leucocidin (PVL) toxin [5].

Management of MRSA infections includes the use of Vancomycin, one of the essential drugs of choice for invasive infections. Other antibiotics, such as Clindamycin, Trimethoprim-sulfamethoxazole and Tetracycline, are also used, with some offered in combination with a beta-lactam such as Amoxicillin or with other antibiotics such as Cefazolin, Linezolid, or Rifampicin [6]. Generally, MRSA strains have been associated with increased incidences of resistance to other antibiotics. This has been attributed not only to the global trend of antibiotic resistance due to over- and indiscriminate use, but also to the unique ability of the pathogen to easily acquire resistance [7–9].

MRSA has been clustered into different epidemiological groups based on the type of SCC*mec* element carriage and the virulence related to the PVL toxin. These include HA-MRSA, CA-MRSA, and livestock-associated-MRSA (LA-MRSA). While CA-MRSA is associated with smaller SCC*mec* elements (types IV and V) and often harbours the potent PVL toxin, HA-MRSA is characterised by a larger SCC*mec* element (I, II, III) and less frequently produces the PVL toxin [10–12]. This toxin can penetrate undamaged skin causing severe infections. It is thought to have evolved from community-associated methicillin susceptible *S. aureus* strains due to the transmission of the *pvl* gene on bacteriophages [11]. Although the reservoir of CA-MRSA is rapidly expanding, compared to nosocomial strains, CA-MRSA strains are more susceptible to a variety of antibiotics and resistant to a few classes, frequently, the beta-lactams and macrolides [12–14]. Livestock-associated MRSA (LA-MRSA), on the other hand, is commonly found in animals with differing genetic backgrounds from those of CA-MRSA and HA-MRSA [15].

MRSA is able to produce one or more staphylococcal enterotoxins (SEs), which are part of the main virulence factors of the pathogen. These toxins possess potent superantigenic activity and are composed of the classical (SEA-SEE) and newer (SEG-SE*l*Y) types [16]. Members of these SEs play a vital role in outbreaks of food poisoning and other infections that are septic-related [16]. Staphylococcal enterotoxins are heat stable and, therefore, are able to thrive and maintain their activity in food previously contaminated with the pathogen. In their review of MRSA as a foodborne pathogen, Wendlandt et al. [17] conclude that CA-MRSA, LA-MRSA, and HA-MRSA can be present in food and food products meant for human consumption. Although there is no evidence of the direct transmission of MRSA from fish to humans, interaction with the aquatic environment as well as their involvement in handling and consumption of marine food, provides a possibility that MRSA contaminated fish can be a source of foodborne infection to humans. Larsen et al. [18] reported one of the first incidences of food animals as a source of human MRSA infection. The researchers identified a new strain of poultry-associated MRSA in humans in Denmark, without any prior exposure to livestock and, therefore, were most likely acquired by eating or handling contaminated poultry meat. The genetic analysis of the strains linked them to imported poultry strains from other European countries.

There is available data on the occurrence and molecular characteristics of MRSA in humans and other animals. Also, in the last decade, investigation of MRSA in food and food-producing animals has received considerable attention. On the contrary, few studies have focused on MRSA in fish [19–29]. The majority of these studies have focused on the prevalence or incidence of the superbug, with no further characterisation. There is, therefore, an information gap with regard to the characteristics of MRSA from fish. Also, over 99% of previous reports of MRSA from fish have focused on the incidence from wild catch compared to the current study, where over 97% of the isolates were from tank cultured edible marine fish. This study, therefore, focused on antimicrobial resistance, virulence profiles, and possible epidemiological types of MRSA isolated from edible marine fish.

## 2. Materials and Methods

### 2.1. Bacterial Isolates

Thirty-three (33) MRSA isolates were characterised in this study. These isolates were recovered from 100 aquaculture and 20 wild marine fish from an earlier study, which represented the first report of the detection of MRSA in marine aquaculture fish in South Africa [29]. Thirty-two (32) isolates were recovered from aquaculture fish while one was isolated from wild catch. Methicillin resistance was previously determined using Cefoxitin and Oxacillin, followed by PCR detection of the *mecA* gene [29]. All strains were maintained in Tryptic Soy Broth (TSB) supplemented with 25% glycerol and stored at −80°C.

### 2.2. Antibiotic Susceptibility Testing

MRSA isolates were recovered from glycerol stocks by plating on nutrient agar and incubating at 37°C for 24 hours. The isolates were subjected to antibiotic susceptibility testing (12 antibiotics belonging to 9 classes) using the disk diffusion assay on Muller Hinton Agar (MHA) in accordance with the guidelines of the Clinical and Laboratory Standards Institute (CLSI) [30]. Colonies from an overnight pure culture were used to prepare a bacterial suspension in sterile normal saline (0.85%), and the turbidity adjusted to 0.5 McFarland standards. The suspension was uniformly streaked on MHA plates using sterile swabs. Various antibiotic disks (Mast diagnostics, UK), including Chloramphenicol (10 *μ*g), Erythromycin (15 *μ*g), Ampicillin (10 *μ*g), Rifampicin (5 *μ*g), Doxycycline (30 *μ*g), Gentamycin (10 *μ*g), Levofloxacin (5 *μ*g), Clindamycin (2 *μ*g), Imipenem (10 *μ*g), Ciprofloxacin (5 *μ*g), Tetracycline (30 *μ*g), and Trimethoprim-sulphamethoxazole (1.25/23.75 *μ*g) were dispensed on inoculated plates using a Mast discs dispenser (Mast diagnostics, UK). These antibiotics were chosen based on their clinical importance in the management of staphylococcal infections in humans and animals. Plates were inoculated in duplicates and incubated at 35°C for 16–18 hours and the diameters of the zones of inhibition measured to the nearest millimetre. Each mean reading was interpreted according to CLSI breakpoints [30, 31].

The disk diffusion test carried out on *S. aureus,* in response to Vancomycin, does not differentiate “susceptible” from “intermediate” responses [30]. All isolates were, therefore, subjected to Vancomycin broth microdilution assay performed in accordance with the guidelines of CLSI [30]. The stock solution of the antibiotic was prepared and transferred to a microtiter plate, and twofold serial dilutions were prepared using a cation-adjusted Mueller–Hinton broth (CAMHB) to achieve an antibiotic concentration, ranging from 0.25 to 128 *μ*g/mL. Bacterial inocula were added to the wells to achieve a final density equivalent to 5 × 10^5^ CFU/mL, and the absorbance was read at 600 nm prior and postincubation at 35°C for 24 hours. Assays were carried out in duplicates. The lowest antibiotic concentration that produced no growth was considered as the MIC and interpreted according to the CLSI breakpoints [30]. *Staphylococcus aureus* ATCC 25923 was used as a quality-control organism.

Multiple antibiotic resistance (MAR) was considered as resistance to ≥3 antibiotics of at least two classes. MAR phenotypes were generated for each MAR-MRSA isolate consisting of all antimicrobials to which a particular isolate was resistant.

### 2.3. Molecular Detection of Antibiotic Resistance Genes (ARGs), SEs, *pvl*, and SCC*mec* Types

DNA was isolated using the boiling method as previously described [32, 33], with slight modifications. Cells from an overnight culture were suspended in 200 *μ*L of sterile distilled water and lysed by boiling at 100°C for 15 minutes in a digital Accu dri‐block (Labnet, Edison, NJ, USA). Cell-free supernatants were obtained following centrifugation at 13,000 ×g for 5 minutes. The presence and integrity of the DNA were validated using agarose gel electrophoresis. The supernatants were used as DNA templates in all PCR reactions.

Based on the phenotypic antibiotic resistance observed, antimicrobial resistance determinants *femA*, *blaZ*, *ermA*, *ermB*, *ermC*, *tetM*, and *tetA* were detected using specific primers (Inqaba Biotech, Pretoria) (Table 1). A multiplex PCR assay was used to detect genes that encoded the production of staphylococcal enterotoxins A, B, C, D, and E [38]. The cycling conditions included the initiation step at 93°C for 15 minutes; 35 cycles of denaturation at 92°C for 40 seconds; 45–55°C for 60 seconds; 72°C for 90 seconds; and a final extension at 72°C for 7 minutes. A conventional PCR assay was used to detect lukS/F-PV genes that encode the PVL S/F bicomponent proteins using the primer pair described by Lina et al. [39]. The cycling conditions were as follows: Initial denaturation at 94°C for 5 minutes; 40 cycles of 94°C for 40 seconds; 52°C for 40 seconds; 72°C for 90 seconds; and a final extension at 72°C for 8 minutes. For the SCC*mec* types, primer pairs described by Zhang et al. [40] were used. Cycling conditions included an initial denaturation step at 94°C for 5 minutes, 40 cycles of 94°C for 40 seconds, 46°C for 40 seconds, and 72°C for 90 seconds. The final extension was done at 72°C for 8 minutes. The oligonucleotide sequences used for the detection of antibiotic resistance genes, staphylococcal enterotoxins, *pvl,* and SCC*mec* types are shown in Table 2. All PCR amplifications were performed in 25 *μ*L reaction mix, each consisting of 5 *μ*L template DNA, 0.5 *μ*L of each oligonucleotide, 12.5 *μ*L of 2X PCR master mix (Bio Labs, New England), and an appropriate volume of nuclease-free water (Bio Labs, New England). The resulting amplicons were resolved by gel electrophoresis in a 1.5% (w/v) agarose gel run at 100 volts for 40 minutes in a 0.5X TAE buffer. The gels were visualised under a UV trans-illuminator (Alliance 4.7 XD-79, Uvitec, Cambridge, UK).

## 3. Results

### 3.1. Antimicrobial Resistance Profile of MRSA

**Figure 1:**
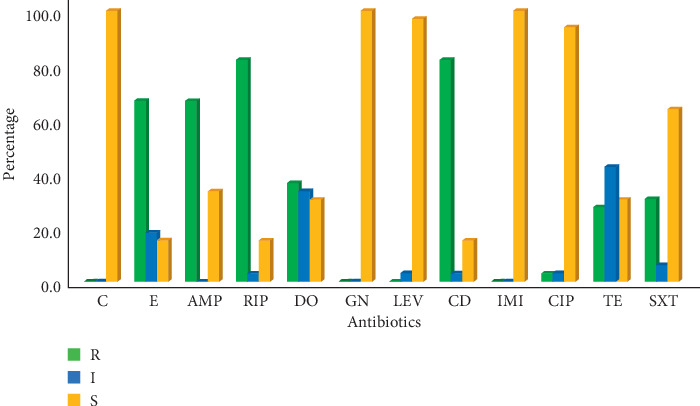
Antimicrobial-resistant patterns of 33 MRSA isolates to 12 antibiotics tested using the disk diffusion assay; C: Chloramphenicol; E: Erythromycin; AMP: Ampicillin; RIP: Rifampicin; DO: Doxycycline; GN: Gentamicin; LEV: Levofloxacin; CD: Clindamycin; IPM: Imipenem; CIP: Ciprofloxacin; TE: Tetracycline, and SXT: Trimethoprim-Sulfamethoxazole.

All MRSA isolates were susceptible to Gentamicin, Chloramphenicol, and Imipenem. On the other hand, the highest antibiotic resistance (82%) was to Rifampicin and Clindamycin, followed by Erythromycin and Ampicillin with 67% (22/33) resistance each. Twelve strains (36%) were resistant to Doxycycline, 10 (30%) to Trimethoprim-Sulfamethoxazole, and 9 (27%) to Tetracycline. Only one (3.0%) strain was resistant to Ciprofloxacin.  Figure 1 shows the resistance profiles of MRSA isolates assayed using the disk diffusion method.

### 3.2. Vancomycin MICs

The MIC for Vancomycin with values of ≤2 *μ*g/mL was recorded in 58% (19/33) of the MRSA strains considered as susceptible, 12% (*n* = 4) with MICs range of 4–8 *μ*g/mL (intermediate), and 30% (*n* = 10) with values of ≥16 *μ*g/mL (resistant).

### 3.3. Multiple Antibiotic Resistance (MAR)

Most (82%, *n* = 27) of the MRSA strains were multiple antibiotic-resistant (resistant to three or more antibiotics of at least 2 classes) with MAR index greater than 0.2. A total of 20 MAR phenotypes were obtained with two strains exhibiting resistance of up to 8 (E-AMP-RIP-DO-CD-VA-TE-SXT) of the 13 antimicrobials tested. The phenotype E-AMP-RIP-CD was dominant (*n* = 4), followed by E-AMP-RIP-DO-CD-VA-SXT (*n* = 3). For all isolates that were resistant to Vancomycin (*n* = 10), at least five other antibiotics were considered ineffective therapies for their in *vitro* inhibition (Table 3).

### 3.4. Prevalence of Antibiotic Resistance Genes

The presence of *femA* genes among MRSA isolates was at a frequency of 85% (28/33), while 59% (13/22) of *blaZ*-positive were recorded from *β*-lactam-resistant strains. The *ermB* gene was present in 7 (32%; 7/22) of Erythromycin-resistant isolates, while *tetM* and *tetA* were recorded in 89% (8/9) and 22% (2/9), respectively, for Tetracycline-resistant strains. Antibiotic resistance determinants *ermA* and *ermC* were not detected in the study.

### 3.5. Occurrence of Enterotoxin Genes, SCC*mec* Types, and *pvl*

Six (18%) isolates were enterotoxigenic, all of which were MDR strains: enterotoxin *e* was detected in 4 strains (12%), followed by *sea* (3%) and *sec* (3.0%) detected in one strain each. A multiplex PCR for SCC*mec* typing revealed a total of 19 (58%) MRSA strains that possessed SCC*mec* types I (*n* = 1), II (*n* = 1) or III (*n* = 17) characteristic of HA-MRSA while 10 (30%) carried the SCC*mec* type IVc element characteristic of CA-MRSA. SCC*mec* type V was not detected and four strains could not be typed. Six (18%) of the 33 MRSA isolates were *pvl* positive distributed as follows: one SCC*mec* type I strain; two SCC*mec* type III strain; and three SCC*mec* type IVc strain.

## 4. Discussion

Antibiotic susceptibility testing revealed Gentamicin, Imipenem, Chloramphenicol, and Ciprofloxacin as the most effective antimicrobials for MRSA, while high resistance to Rifampicin, Clindamycin, Ampicillin, and Erythromycin were observed. Over 80% of the samples were recorded as MDR strains. The high individual resistance to certain antimicrobials and the prevalence of MDR strains are not surprising as multidrug resistance is becoming a typical feature of MRSA, coupled with global antimicrobial resistance challenge [9, 41, 42]. The results obtained in this study are comparable with high levels of up to 100% MDR-MRSA recorded from pigs and retail food in China [43] as well as from poultry and farm workers in South Africa [44]. However, a 37.2% (29/78) prevalence of multidrug-resistant MRSA from retail meat in the United States is in contrast to our findings [45]. High individual resistances of MRSA to Rifampicin (82%), Clindamycin (82%), Erythromycin (67%), and Ampicillin 67% are similar to those recorded by Sato et al. [46], although the percent Tetracycline resistance in our study was much lower (27%) than previously reported (100%) from pigs in Japan [46]. The resistance of isolates to Vancomycin was 30%. This is quite disturbing as Vancomycin represents the standard therapy for invasive MRSA infections in humans [47, 48]. Vancomycin-resistant MRSA has also been detected in camel meat, whereby all isolates detected as Vancomycin-resistant *S. aureus* were MRSA strains [49]. Other studies have revealed the emergence of Vancomycin-resistant MRSA strains in other parts of the world, since its first detection in 1996 in a hospital case in Japan, and in the US in 2002 [47, 50]. The increased frequency of resistance of MRSA to this antibiotic suggests the need for combination therapy or adoption of alternatives or inclusion of newer regimens for the treatment of invasive MRSA infections.

Antibiotic resistance determinants were prevalent in MRSA isolates and included the following: *femA*, *blaZ*, *tetA*, *tetM*, and *ermB*. The chromosomally located *femA* gene has been shown to encode proteins that significantly affect the level of Methicillin resistance in *S. aureus* thus, probably the reason for its high prevalence in the MRSA strains in this study. PCR detection of this gene, in addition to *mecA* detection, has been proposed as a reliable indicator of MRSA that easily differentiates it from *mecA*-positive coagulase-negative staphylococci [51]. Resistance genes, such as *tetA*, *tetM,* and *ermB* reported from our isolates, have also been reported in MRSA isolated from poultry and farm workers in South Africa [44] while *tetM* and *ermB* were reported in clinical isolates [52]. More antibiotic-resistant bacterial strains are continually reported due to increased use of the same or similar antibiotics in aquaculture, animal husbandry, and in the management of human diseases. Also, large-scale settings of aquaculture have led to increased antibiotic resistance in bacteria that are potentially pathogenic to both fish and humans [53].

One of the most common seafood-borne intoxications is due to preformed SEs in food, thus resulting in outbreaks of foodborne diseases around the world. Of these, staphylococcal enterotoxin A, followed by D, is the most frequently associated with these outbreaks, although the other classical type toxins, together with SEH, have also been reported [54]. These toxins are also involved in toxic shock syndrome and other staphylococcal infections. In the present study, a total of 6 MRSA strains were recorded as positive for genes encoding the classical type SEs, with *see* detected in four of the strains. These results are contrary to other studies with higher frequencies of enterotoxigenic MRSA [55–57]. However, this is the first report that focused on the detection of toxin genes in MRSA strains isolated from marine fish in South Africa. The most frequently detected gene was *see*, which is in line with the study by Arfatahery et al. [57]. Growth of *Staphylococcus* sp. and production of enterotoxin in food have been reported to be aided by poor personal hygiene, inadequate refrigeration, delays in processing, and postprocess contamination [54]. The low levels of these enterotoxins in the present study, if not intrinsic, could be due to minimal delay time between sample collection and analysis. Just a few handlers were in contact with the fish samples. However, continuous food monitoring is still required to minimise contamination with enterotoxigenic MRSA that can occur between fish harvesting, throughout processing to the final product.

The types of SCC*mec* detected were I, II, III, and IV although I and II were detected at a relatively lower frequency to types III (*n* = 17) and IV (*n* = 10). This is contrary to the findings where the bulk of SCC*mec* from MRSA isolates detected from processed food in Pakistan were type IV followed by types II and III [58]. Type IV MRSA strains have also been isolated from retail meat and humans in Georgia [59]. Studies have shown that SCC*mec* types I-III are mostly associated with HA-MRSA, while types IV and V are linked to CA-MRSA [10–12, 60, 61]. The results, therefore, are an indication that over 57% of strains in the current study were likely to be HA-MRSA while 30% were CA-MRSA. This is an indication that fish is a potential reservoir for HA- and CA-MRSA to humans. Four strains could not be typed and were considered to belong to other SCC*mec* types/subtypes not included in the screen.

The PVL toxin has gained considerable attention as one of the major virulence factors present in CA-MRSA strains [12, 13, 41, 62]. Consequently, it has been used as an epidemiological tool to determine the nature of MRSA, whether it is CA-MRSA or HA-MRSA. A low frequency of *pvl* (18.2%; 6/33) was recorded in this study, although higher than studies which revealed the absence of the gene in isolates from retail raw fish and raw meat samples [24, 56]. This gene is mostly associated with CA-MRSA strain, which is reflected in the study as 16% (3/19) of HA-MRSA possessed the *pvl* gene compared to 30% (3/10) of CA-MRSA strains. Sivaraman et al. [63] also found the *pvl* gene in approximately 16% of CA-MRSA from seafood in India, although lower than the percentage recorded in this study.

## 5. Conclusions

This study revealed a wide range of multiple antibiotic-resistance profiles of MRSA isolated from marine fish. Although the prevalence of toxin genes was low, food poisoning from infection with such strains cannot be ruled out. Thus, there is a need for continuous and better control of sources of food contamination and the spread of antimicrobial-resistant bacteria since the pathogenic potential of these strains cannot be ignored.

## Figures and Tables

**Table 1:** Primer sequences used for the detection of antibiotic resistance genes.

Target gene	Oligonucleotide sequences (5′-3′)	Product size (bp)	Annealing temperature (°C)	References

*ermA*	F: TATCTTATCGTTGAGAAGGGATT	139	47.6	
	R: CTACACTTGGCTTAGGATGAAA			
*ermB*	F: CTATCTGATTGTTGAAGAAGGATT	142	47	[34]
	R: GTTTACTCTTGGTTTAGGATGAAA			
*ermC*	F: CTTGTTGATCACGATAATTTCC	190		
	R: ATCTTTTAGCAAACCCGTATTC			
*blaZ*	F: ACTTCAACACCTGCTGCTTTC	173	49	
	R: TGACCACTTTTATCAGCAACC			

*tetA*	F: GCTACATCCTGCTTGCCTTC	209	50	[35]
	R: ATAGATCGCCGTGAAGAGG			

*tetM*	F: AGTGGAGCGATTACAGAA	158	45	[36]
	R: CAT ATG TCC TGG CGT GTC TA			

*femA*	F: AAAAAAGCACATAACAAGCG	132	45.5	[37]
	R: GATAAAGAAGAAACCAGCAG			

**Table 2:** Oligonucleotide sequences used for the detection of staphylococcal enterotoxins, *pvl*, and SCC*mec* types.

Gene target/specificity	Sequence (5′-3′)	Product size (bp)	Reference

Universal fw	F: TGTATGTATGGAGGTGTAAC	—	[38]
*Sea*	R: ATTAACCGAAGGTTCTGT	270	
*Seb*	R: ATAGTGACGAGTTAGGTA	165	
*Sec*	R: AAGTACATTTTGTAAGTTCC	69	
*Sed*	R: TTCGGGAAAATCACCCTTAA	306	
*See*	R: GCCAAAGCTGTCTGAG	213	

Luk-PV*L*	F: ATCATTAGGTAAAATGTCTGGACATGATCCA	433	[39]
	R: GCATCAAGTGTATTGGATAGCAAAAGC		

SCC*mec* I	F: GCTTTAAAGAGTGTCGTTACAGG	613	[40]
	R: GTTCTCTCATAGTATGACGTCC		
SCC*mec* II	F: CGTTGAAGATGATGAAGCG	398	
	R: CGAAATCAATGGTTAATGGACC		
SCC*mec* III	F: CCATATTGTGTACGATGCG	280	
	R: CCTTAGTTGTCGTAACAGATCG		
SCC*mec* Iva	F: GCCTTATTCGAAGAAACCG	776	
	R: CTACTCTTCTGAAAAGCGTCG		
SCC*mec* IVb	F: TCTGGAATTACTTCAGCTGC	493	
	R: AAACAATATTGCTCTCCCTC		
SCC*mec* IVc	F: ACAATATTTGTATTATCGGAGAGC	200	
	R: TTGGTATGAGGTATTGCTAAAG		
SCC*mec* IVd	F: CTCAAAATACGGACCCCAATACA	881	
	R: TGCTCCAGTAATTGCTAAAG		
SCC*mec* V	F: GAACATTGTTACTTAAATGAGCG	325	
	R: TGAAAGTTGTACCCTTGACACC		

**Table 3:** Phenotypes of MDR-MRSA isolates.

No. of antibiotics resistant	Resistant phenotype	Frequency of occurrence

3	AMP-RIP-CD	1
	E-RIP-CD	1
	E-DO-TE	1
	E-RIP-DO	1
	DO-CD-TE	1
4	DO-CD-TE-SXT	1
	E-AMP-RIP-CD	1
	AMP-RIP-DO-CD	1
	AMP-RIP-CD-TE	1
	E-AMP-RIP-CD	4
	E-RIP-CD-SXT	1
	E-AMP-RIP-CD	1
5	E-AMP-RIP-CD-SXT	1
6	E-AMP-RIP-CD-VA-TE	1
	E-AMP-RIP-CD-VA-SXT	1
	E-AMP-RIP-DO-CD-TE	1
	E-AMP-RIP-CD-VA-SXT	2
7	E-AMP-RIP-CD-VA-CIP-TE	1
	E-AMP-RIP-DO-CD-VA-SXT	3
8	E-AMP-RIP-DO-CD-VA-TE-SXT	2

C: Chloramphenicol; E: Erythromycin; AMP: Ampicillin; RIP: Rifampicin; DO: Doxycycline; GN: Gentamicin; LEV: Levofloxacin; CD: Clindamycin; IPM: Imipenem; CIP: Ciprofloxacin; TE: Tetracycline, and SXT: Trimethoprim-Sulfamethoxazole.

## Data Availability

The data used to support the findings of this study are included in the article.

## References

[1] Tsubakishita S., Kuwahara-Arai K., Baba T., Hiramatsu K. (2010). Staphylococcal cassette chromosome *mec*-like element in *Macrococcus caseolyticus*. *Antimicrobial Agents and Chemotherapy*.

[2] Lee A. S., de Lencastre H., Garau J. (2018). Methicillin-resistant *Staphylococcus aureus*. *Nature Reviews Disease Primers*.

[3] Dyke K. G. H., Jevons M. P., Parker M. T. (1966). Penicillinase production and intrinsic resistance to penicillins in *Staphylococcus aureus*. *The Lancet*.

[4] F Chambers H., DeLeo F. R. (2009). Waves of resistance: *Staphylococcus aureus* in the antibiotic era. *Nature Reviews Microbiology*.

[5] Turlej A., Hryniewicz W., Empel J. (2011). Staphylococcal cassette chromosome *mec* (SCC*mec*) classification and typing methods: an overview. *Polish Journal Microbiology*.

[6] Liu C., Bayer A., Cosgrove S. E. (2011). Clinical practice guidelines by the infectious diseases society of America for the treatment of methicillin-resistant *Staphylococcus aureus* infections in adults and children. *Clinical Infectious Diseases*.

[7] Grundmann H., Aires-de-Sousa M., Boyce J., Tiemersma E. (2006). Emergence and resurgence of meticillin-resistant *Staphylococcus aureus* as a public-health threat. *The Lancet*.

[8] Onanuga A., Awhowho G. O. (2012). Antimicrobial resistance of *Staphylococcus aureus* strains from patients with urinary tract infections in Yenagoa, Nigeria. *Journal of Pharmacy and Bioallied Sciences*.

[9] Poovelikunnel T. T., Budri P. E., Shore A. C., Coleman D. C., Humphreys H., Fitzgerald-Hughes D. (2018). Molecular characterization of nasal methicillin-resistant *Staphylococcus aureus* isolates showing increasing prevalence of mupirocin resistance and associated multidrug resistance following attempted decolonization. *Antimicrobial Agents and Chemotherapy*.

[10] Vandenesch F., Naimi T., Enright M. C. (2003). Community-acquired methicillin-resistant *Staphylococcus aureus* carrying panton-valentine leukocidin genes: worldwide emergence. *Emerging Infectious Diseases*.

[11] Gülmez D., Sancak B., Ercis S., Karakaya J., Hascelik G. (2012). Investigation of SCC*mec* types and panton-valentine leukocidin in community-acquired and nosocomial *Staphylococcus aureus* strains: comparing skin and soft tissue infections to the other infections. *Mikrobiyoloji Bulteni*.

[12] Ono H. K., Sato’o Y., Narita K. (2015). Identification and characterization of a novel staphylococcal emetic toxin. *Applied and Environmental Microbiology*.

[13] Appelbaum P. C. (2007). Microbiology of antibiotic resistance in *Staphylococcus aureus*. *Clinical Infecious Diseases*.

[14] Maree C. L., Daum R. S., Boyle-Vavra S., Matayoshi K., Miller L. G. (2007). Community-associated methicillin-resistant *Staphylococcus aureus* isolates and healthcare-associated infections. *Emerging Infectious Diseases*.

[15] Li S., Skov R. L., Han X. (2011). Novel types of staphylococcal cassette chromosome *mec* elements identified in clonal complex 398 methicillin-resistant *Staphylococcus aureus* strains. *Antimicrobial Agents and Chemotherapy*.

[16] Fisher E. L., Otto M., Cheung G. Y. C. (2018). Basis of virulence in enterotoxin-mediated staphylococcal food poisoning. *Frontiers in Microbiology*.

[17] Wendlandt S., Schwarz S., Silley P. (2013). Methicillin-resistant *Staphylococcus aureus*: a food-borne pathogen?. *Annual Review of Food Science and Technology*.

[18] Larsen J., Stegger M., Andersen P. S. (2016). Evidence for human adaptation and foodborne transmission of livestock-associated methicillin-resistant *Staphylococcus aureus*. *Clinical Infectious Diseases*.

[19] Ayulo A. M. R., Machado R. A., Scussel V. M. (1994). Enterotoxigenic *Escherichia coli* and *Staphylococcus aureus* in fish and seafood from the southern region of Brazil. *International Journal of Food Microbiology*.

[20] Albuquerque W. F., Macrae A., Sousa O. V., Vieira G. H. F., Vieira R. H. S. F. (2007). Multiple drug resistant *Staphylococcus aureus* strains isolated from a fish market and from fish handlers. *Brazilian Journal of Microbiology*.

[21] Boer E. D., Zwartkruis-Nahuis J. T. M., Wit B. (2009). Prevalence of methicillin-resistant *Staphylococcus aureus* in meat. *International Journal of Food Microbiology*.

[22] Atyah M. A. S., Zamri-Saad M., Siti-Zahrah A. (2010). First report of methicillin-resistant *Staphylococcus aureus* from cage-cultured tilapia (*Oreochromis niloticus*). *Veterinary Microbiology*.

[23] Beleneva I. A. (2011). Incidence and characteristics of *Staphylococcus aureus* and *Listeria monocytogenes* from the Japan and South China seas. *Marine Pollution Bulletin*.

[24] Hammad A. M., Watanabe W., Fujii T., Shimamoto T. (2012). Occurrence and characteristics of methicillin-resistant and-susceptible *Staphylococcus aureus* and methicillin-resistant coagulase-negative staphylococci from Japanese retail ready-to-eat raw fish. *International Journal of Food Microbiology*.

[25] Cho J. I., Joo I. S., Choi J. H. (2014). Distribution of methicillin-resistant *Staphylococcus aureus* (MRSA) in RAW meat and fish samples in Korea. *Food Science and Biotechnology*.

[26] Obaidat M. M., Bani Salman A. E., Lafi S. Q. (2015). Prevalence of *Staphylococcus aureus* in imported fish and correlations between antibiotic resistance and enterotoxigenicity. *Journal of Food Protection*.

[27] Chajęcka-Wierzchowska W., Zadernowska A., Nalepa B., Sierpińska M., Łaniewska-Trokenheim Ł. (2015). Coagulase-negative staphylococci (CoNS) isolated from ready-to-eat food of animal origin–phenotypic and genotypic antibiotic resistance. *Food Microbiology*.

[28] Visnuvinayagam S., Joseph T., Murugadas V., Chakrabarti R., Lalitha K. V. (2015). Status on methicillin resistant and multiple drug resistant *Staphylococcus aureus* in fishes of Cochin and Mumbai coast, India. *Journal of Environmental Biology*.

[29] Fri J., Ndip R. N., Njom H. A., Clarke A. M. (2018). First report of methicillin‐resistant *Staphylococcus aureus* in tank cultured dusky kob (*Argyrosomus japonicus*), and evaluation of three phenotypic methods in the detection of MRSA. *Journal of Food Safety*.

[30] Clinical and Laboratory Standards Institute (CLSI) (2016). *Performance Standards for Antimicrobial Susceptibility Testing Twenty-Fourth Informational Supplement (M100S)*.

[31] Clinical and Laboratory Standards Institute (CLSI) (2012). *Performance Standards for Antimicrobial Susceptibility Testing Twenty-Second Informational Supplement (M100-S22)*.

[32] Oliveira C. F. D., Paim T. G. D. S., Reiter K. C., Rieger A., D’azevedo P. A. (2014). Evaluation of four different DNA extraction methods in coagulase-negative staphylococci clinical isolates. *Revista do Instituto de Medicina Tropical de São Paulo*.

[33] Junior J. C. R., Tamanini R., Soares B. F. (2016). Efficiency of boiling and four other methods for genomic DNA extraction of deteriorating spore-forming bacteria from milk. *Semina: Ciências Agrárias*.

[34] Martineau F., Picard F. J., Lansac N. (2000). Correlation between the resistance genotype determined by multiplex PCR assays and the antibiotic susceptibility patterns of *Staphylococcus aureus* and *Staphylococcus epidermidis*. *Antimicrobial Agents and Chemotherapy*.

[35] Ng L.-K., Martin I., Alfa M., Mulvey M. (2001). Multiplex PCR for the detection of tetracycline resistant genes. *Molecular and Cellular Probes*.

[36] Strommenger B., Kettlitz C., Werner G., Witte W. (2003). Multiplex PCR assay for simultaneous detection of nine clinically relevant antibiotic resistance genes in *Staphylococcus aureus*. *Journal of Clinical Microbiology*.

[37] Mehrotra M., Wang G., Johnson W. M. (2000). Multiplex PCR for detection of genes for *Staphylococcus aureus* enterotoxins, exfoliative toxins, toxic shock syndrome toxin 1, and methicillin resistance. *Journal of Clinical Microbiology*.

[38] Sharma N. K., Rees C. E. D., Dodd C. E. R. (2000). Development of a single-reaction multiplex PCR toxin typing assay for *Staphylococcus aureus* strains. *Applied Environmental Microbiology*.

[39] Lina G., Piemont Y., Godail-Gamot F. (1999). Involvement of panton-valentine leukocidin—producing *Staphylococcus aureus* in primary skin infections and pneumonia. *Clinical Infectious Diseases*.

[40] Zhang K., McClure J.-A., Elsayed S., Louie T., Conly J. M. (2005). Novel multiplex PCR assay for characterization and concomitant subtyping of staphylococcal cassette chromosome *mec* types I to V in methicillin-resistant *Staphylococcus aureus*. *Journal of Clinical Microbiology*.

[41] Diep B. A., Chambers H. F., Graber C. J. (2008). Emergence of multidrug-resistant, community-associated, methicillin-resistant *Staphylococcus aureus* clone USA300 in men who have sex with men. *Annals of Internal Medicine*.

[42] Gonzales P. R., Pesesky M. W., Bouley R. (2015). Synergistic, collaterally sensitive *β*-lactam combinations suppress resistance in MRSA. *Nature Chemical Biology*.

[43] Wang W., Liu F., Baloch Z. (2017). Genotypic characterization of methicillin-resistant *Staphylococcus aureus* isolated from pigs and retail foods in China. *Biomedical and Environmental Sciences*.

[44] Amoako D. G., Somboro A. M., Abia A. L. K. (2019). Genomic analysis of methicillin-resistant *Staphylococcus aureus* isolated from poultry and occupational farm workers in Umgungundlovu district, South Africa. *Science of the Total Environment*.

[45] Ge B., Mukherjee S., Hsu C.-H. (2017). MRSA and multidrug-resistant *Staphylococcus aureus* in US retail meats, 2010–2011. *Food Microbiology*.

[46] Sato T., Usui M., Motoya T., Sugiyama T., Tamura Y. (2015). Characterisation of methicillin-resistant *Staphylococcus aureus* ST97 and ST5 isolated from pigs in Japan. *Journal of Global Antimicrobial Resistance*.

[47] Hiramatsu K. (2001). Vancomycin-resistant *Staphylococcus aureus*: a new model of antibiotic resistance. *The Lancet Infectious Diseases*.

[48] Drew R. H. (2007). Emerging options for treatment of invasive, multidrug‐resistant *Staphylococcus aureus* infections. *Pharmacotherapy*.

[49] Al-Amery K., Elhariri M., Elsayed A. (2019). Vancomycin-resistant *Staphylococcus aureus* isolated from camel meat and slaughterhouse workers in Egypt. *Antimicrobial Resistance and Infection Control*.

[50] Howden B. P., Ward P. B., Charles P. G. (2004). Treatment outcomes for serious infections caused by methicillin-resistant *Staphylococcus aureus* with reduced vancomycin susceptibility. *Clinical Infectious Diseases*.

[51] Ali A. (2016). Detection of *mecA*, *mecC* and *femB* genes by multiplex polymerase chain reaction. *Journal of Veterinary Advances*.

[52] Duran N., Ozer B., Duran G. G., Onlen Y., Demir C. (2012). Antibiotic resistance genes and susceptibility patterns in staphylococci. *The Indian Journal of Medical Research*.

[53] Alcaide E., Blasco M.-D., Esteve C. (2005). Occurrence of drug-resistant bacteria in two European eel farms. *Applied Environmental Microbiology*.

[54] Argudín M. Á., Mendoza M. C., Rodicio M. R. (2010). Food poisoning and *Staphylococcus aureus* enterotoxins. *Toxins*.

[55] Holeckova B., Holoda E., Fotta M., Kalináčová V., Gondol J., Grolmus J. (2002). Occurrence of enterotoxigenic *Staphylococcus aureus* in food. *Annals of Agricultural and Environmental Medicine*.

[56] Rhee C. H., Woo G.-J. (2010). Emergence and characterization of foodborne methicillin-resistant *Staphylococcus aureus* in Korea. *Journal of Food Protection*.

[57] Arfatahery N., Davoodabadi A., Abedimohtasab T. (2016). Characterization of toxin genes and antimicrobial susceptibility of *Staphylococcus aureus* isolates in fishery products in Iran. *Scientific Reports*.

[58] Mirani Z. A., Aqeel A., Naz S., Siddiqi A., Khan M. N., Khan S. I. (2017). Prevalence of staphylococci in commercially processed food products in Karachi-Pakistan. *Journal of Microbiology and Infectious Diseases*.

[59] Jackson C. R., Davis J. A., Barrett J. B. (2013). Prevalence and characterization of methicillin-resistant *Staphylococcus aureus* isolates from retail meat and humans in Georgia. *Journal of Clinical Microbiology*.

[60] Japoni A., Jamalidoust M., Farshad S. (2011). Characterization of SCC*mec* types and antibacterial susceptibility patterns of methicillin-resistant *Staphylococcus aureus* in Southern Iran. *Japanese Journal of Infectious Diseases*.

[61] Valsesia G., Rossi M., Bertschy S., Pfyffer G. E. (2010). Emergence of SCC*mec* type IV and SCC*mec* type V methicillin-resistant *Staphylococcus aureus* containing the panton-valentine leukocidin genes in a large academic teaching hospital in central Switzerland: external invaders or persisting circulators?. *Journal of Clinical Microbiology*.

[62] Lo W.-T., Wang C.-C. (2011). Panton-valentine leukocidin in the pathogenesis of community-associated methicillin-resistant *Staphylococcus aureus* infection. *Pediatrics and Neonatology*.

[63] Sivaraman G. K., Deesha V., Prasad V. (2016). Incidence of community acquired methicillin-resistant *Staphylococcus aureus* (CA-MRSA) in seafood and its environment, Gujarat, India. *International Journal of Scientific Research and Management Studies*.

